# Identification of a Novel Mutation in the PAH Gene in an Iranian Phenylketonuria Family: A Case Report

**Published:** 2017-04

**Authors:** Masoumeh RAZIPOUR, Daniz KOOSHAVAR, Elaheh ALAVINEJAD, Seyede Zahra SAJEDI, Neda MOHAJER, Aria SETOODEH, Saeed TALEBI, Mohammad KERAMATIPOUR

**Affiliations:** 1. Dept. of Medical Genetics, School of Medicine, Tehran University of Medical Sciences, Tehran, Iran; 2. Dept. of Medical Genetics, School of Medicine, Tabriz University of Medical Sciences, Tabriz, Iran; 3. Immunology Research Center, Tabriz University of Medical Sciences, Tabriz, Iran; 4. Dept. of Endocrinology, Children’s Hospital Medical Center, Tehran University of Medical Sciences, Tehran, Iran

**Keywords:** Phenylketonurias, Phenylalanine hydroxylase, Mutation analysis, Iran

## Abstract

Phenylketonuria (PKU) is an inborn error of amino acid metabolism with an autosomal recessive inheritance caused in most cases by mutations in the phenylalanine hydroxylase (PAH) gene. PKU has wide allelic heterogeneity. Here we report a novel heterozygous substitution (c.1223G>T (p.Arg408Leu)) in the PAH gene in an Iranian PKU family. The patient was 19-yr-old female with diagnosis of moderate PKU referred to Department of Medical Genetics, Tehran University of Medical Sciences, Tehran, Iran for genetic counseling/analysis in April 2015. We used PCR-Sequencing to identify any sequence variations in the PAH gene.

## Introduction

Phenylketonuria (PKU; OMIM 261600) is the most common inborn error of amino acid metabolism with an autosomal recessive inheritance (1–3). The incidence of PKU is about 1/10000 in Caucasians (3), but it is thought that the incidence of PKU in Iranian population is more than 1/10000 due to the high prevalence of consanguinity (4–8). In about 98% of cases, hyperphenylalaninaemia (HPA) is due to loss-of-function mutations in PAH gene coding the hepatic enzyme phenylalanine hydroxylase (PAH) (1–3). Absence or deficiency of PAH enzyme causes the elevation of phenylalanine (Phe) and its metabolic derivatives in body fluids and this may result in severe and irreversible mental retardation (4).

The human PAH gene is located on the long arm of chromosome 12 (12q23.2). It contains 13 exons and 12 introns and spans about 90 kbp encoding a monomer of 452 amino acids (1, 9–12). The PAH gene contains different polymorphic markers consisting of a variable number of tandem repeats (VNTRs) of 30-bp cassettes, a series of short (tetranucleotide, (TCTA)_n_) tandem repeats (STR), eight biallelic restriction fragment length polymorphisms (RFLPs) and single nucleotide polymorphisms (SNPs). Combination of these polymorphic markers can generate extended PAH-locus haplotypes. However two multiallelic markers (STR & VNTR) can be used for minihaplotype analysis of PAH region (13, 14).

Given the high allelic heterogeneity in PKU, the spectrum of mutations is different in various populations around the world. Thus mutation analysis of PKU patients is crucial in every population. Here we represent the detailed data about the detection of a novel variation in the PAH gene in an investigation of an Iranian family with PKU.

## Case report

A 19-yr-old female with diagnosis of moderate PKU was referred to Department of Medical Genetics, Tehran University of Medical Sciences, Tehran, Iran to determine the causing mutations in PAH gene in April 2015. Tetrahydrobiopterin (BH4) loading test was performed and she was BH4-nonresponsive.

Informed consent was taken from the patient and her parents. The Ethics Committee of the university approved the study.

PCR-Sequencing of 13 exons of the PAH gene and their flanking intron regions was used to identify any sequence variations in the patient. After mutation detection in patient, carrier status was confirmed for parents. Haplotype analysis of PAH region in this family was performed using fragment analysis by capillary electrophoresis (Pishgam Biotech Co., Tehran, Iran).

**Fig. 1:**
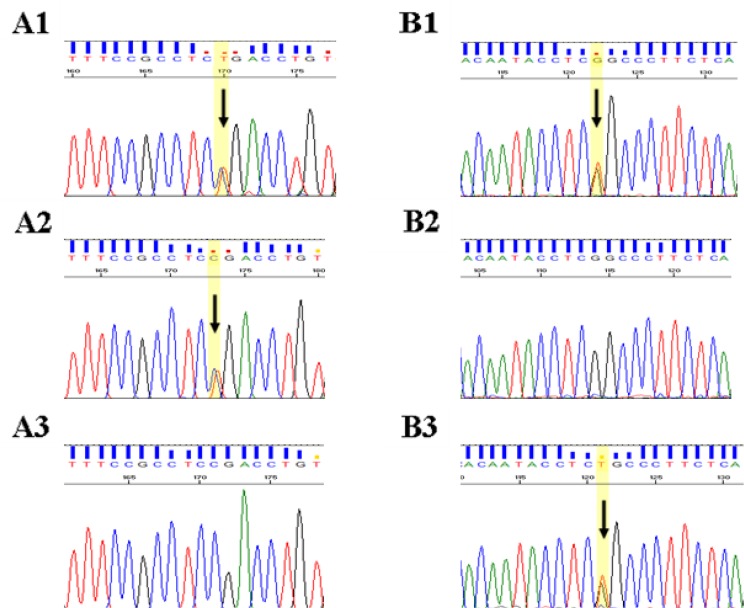
**A:** Nonsense mutation (c.727C>T) **A1:** Affected (Heterozygote) **A2:** Father (Heterozygote) **A3:** Mother (Normal). **B:** Novel nucleotide variation (c.1223G>T) **B1:** Affected (Heterozygote) **B2:** Father (Normal) **B3:** Mother (Heterozygote)

**Fig. 2:**
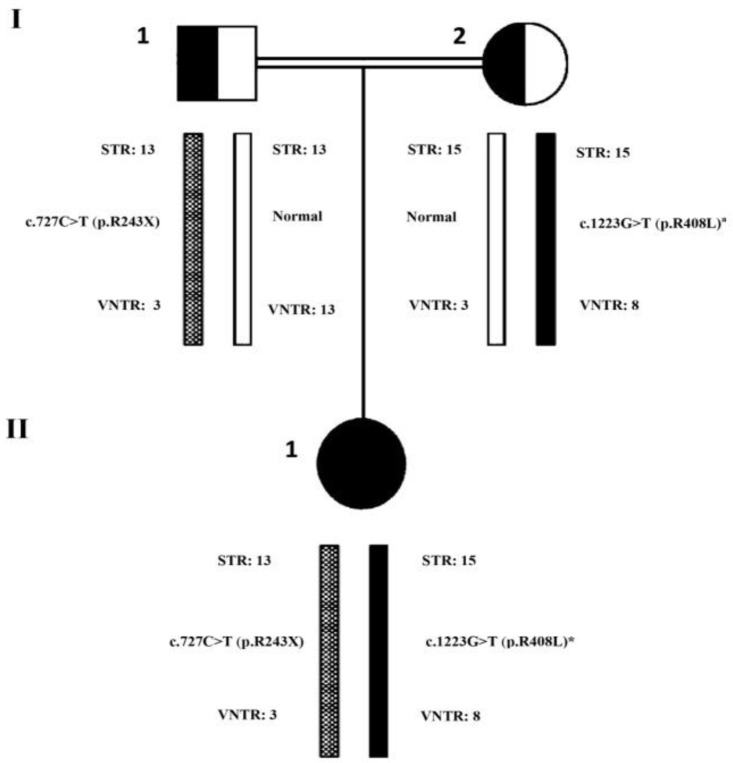
Segregation analysis of mutations and minihaplotypes at the PAH gene in family with the novel variation. ^a^ Novel variation

The patient was compound heterozygote for two variations in exons 7 & 12 (Table 1). Fig. 1 demonstrates sequence chromatograms of variations in exons 7 & 12 of PAH gene in this family. Minihaplotype analysis of PAH region determined the patient and her parents haplotypes as shown in (Fig. 2).

**Table 1:** The mutations found in the PAH gene in a 19-yr-old phenylketonuria patient from Iran

**Variant genotype**^**a**^	**Amino acid change**^**a**^	**Location**	**Mutation type**	**Homo/Hetero**
c.1223G>T^b^	p.Arg408Leu^b^	Exon 12	Missense	Hetero
c.727C>T	p.Arg243Ter	Exon7	Nonesense	Hetero

a HGVS (Human Genome Variation Society) nomenclature

b Novel variation

According to our survey, the variation, c.1223G>T (p.Arg408Leu), has not been previously reported on PAHdb, db SNP, PubMed and HGMD^®^ Professional. Bioinformatic investigation was performed using online tools including PolyPhen-2, SIFT, Provean and Mutation Taster to predict the possible effect of this novel missense variation on the function of PAH protein. The variation was predicted with high confidence to be “disease causing”, “deleterious”, “damaging” and “probably damaging” by Mutation Taster, Provean (score= −5.911), SIFT (score=0), and PolyPhen-2 (score=0.999), respectively (Table 2).

**Table 2:** The results of bioinformatic investigations

**Amino acid change**	**Polyphen-2 prediction**		**SIFT prediction**		**Provean prediction**		**Mutation taster prediction**	
p.Arg408Leu	Score (0–1)	Prediction	Score	Prediction (cutoff: 0.05)	Score	Prediction (cutoff: −2.5)	Score (0–215)	Prediction
	0.999	Probably damaging	0	Damaging	−5.911	Deleterious	102	Disease causing

Furthermore, phylogenetic comparison was performed to show the conservation of the Arg408-PAH residue using the consurf server (Table 3). We found that Arg408-PAH is considered a phylogenetically conserved residue.

**Table 3:** Conservation status of position of the Arg408-PAH residue

**Protein**	**Position**	**Amino acid**	**Conservation score^a^ (Scale^b^)**	**MSA data^c^**	**Residue variety**
PAH	408	Arg	−0.638 (8)	150/150	Lys, Arg

a The normalized conservation scores.

b Scale representing the conservation scores (9 – conserved, 1 – variable).

c The number of aligned sequences having an amino acid (non-gapped) from the overall number of sequences at each position

## Discussion

Here we report the characterization of a novel PAH variation in an Iranian patient with moderate PKU. The novel missense variation is characterized by a G>T transition leading to the substitution of Arg (CGG) with Leu (CTG) in the codon 408 in exon 12 of the PAH gene. Arg408 is a phylogenetically conserved residue in the PAH protein and it is one of the most important residues at the interface between the catalytic domain and the tetramerization domain. Arg408 is located in hinge loop which connects tetramerization domain arm to catalytic domain and H-bonds to Leu311 and Leu308 carbonyl-oxygens (11, 15).

In this position, two other mutations have been previously reported as a disease causing mutation in PKU, including the p.Arg408Trp (c.1222C>T) and p.Arg408Gln (c.1223G>A) substitutions (13). p.Arg408Trp mutation (the most frequent mutation in the PAHdb) disrupts important hydrogen bonds in hinge loop between the catalytic domain and the tetramerization domain and results in a severe metabolic PKU phenotype and causes a low (<1 to <2.7%) residual activity of PAH enzyme (11, 15). p.Arg408Gln mutation decreases dimer stability of PAH protein and is associated with non-PKU HPA (mild HPA) phenotype which is responsive to BH4 (16). In the present study, we identified a novel p.Arg408Leu (c.1223G>T) variation in the same position that results in a basic polar residue change to a nonpolar one. According to our bioinformatics survey, this novel variation can have deleterious and damaging impact on the function of the PAH protein due to its possible effect on hydrogen bonds in hinge loop between the catalytic domain and the tetramerization domain of the PAH protein. Thus it can be considered the mutation which caused the disease in this patient in a compound heterozygous status together with the previously known c.727C>T mutation.

## Conclusion

In this patient, the novel variation (p.Arg408Leu (c.1223G>T)) was associated with a moderate variant of PKU which was not BH4-responsive. Besides, population and in vitro expression analyses are required to show the accurate pathological impact of this variation.

## Ethical considerations

Ethical issues (Including plagiarism, Informed Consent, misconduct, data fabrication and/or falsification, double publication and/or submission, redundancy, etc) have been completely observed by the authors.
